# Effects of Oats, Tartary Buckwheat, and Foxtail Millet Supplementation on Lipid Metabolism, Oxido-Inflammatory Responses, Gut Microbiota, and Colonic SCFA Composition in High-Fat Diet Fed Rats

**DOI:** 10.3390/nu14132760

**Published:** 2022-07-04

**Authors:** Yong Wang, Wentao Qi, Xiaoxuan Guo, Ge Song, Shaojie Pang, Wei Fang, Zhenzhen Peng

**Affiliations:** 1 Academy of National Food and Strategic Reserves Administration, Beijing 100037, China; wangy@ags.ac.cn (Y.W.); sg@ags.ac.cn (G.S.); psj@ags.ac.cn (S.P.); fw@ags.ac.cn (W.F.); 13974364725@163.com (Z.P.); 2 Institute of Quality Standard and Testing Technology for Agro-Products, Chinese Academy of Agricultural Sciences, Beijing 100081, China; guoxiaoxuan@caas.cn

**Keywords:** coarse cereals, lipid metabolism, oxido-inflammatory responses, gut microbiota, short-chain fatty acids

## Abstract

Coarse cereals rich in polyphenols, dietary fiber, and other functional components exert multiple health benefits. We investigated the effects of cooked oats, tartary buckwheat, and foxtail millet on lipid profile, oxido-inflammatory responses, gut microbiota, and colonic short-chain fatty acids composition in high-fat diet (HFD) fed rats. Rats were fed with a basal diet, HFD, oats diet (22% oat in HFD), tartary buckwheat diet (22% tartary buckwheat in HFD), and foxtail millet diet (22% foxtail millet in HFD) for 12 weeks. Results demonstrated that oats and tartary buckwheat attenuated oxidative stress and inflammatory responses in serum, and significantly increased the relative abundance of *Lactobacillus* and *Romboutsia* in colonic digesta. Spearman’s correlation analysis revealed that the changed bacteria were strongly correlated with oxidative stress and inflammation-related parameters. The concentration of the butyrate level was elevated by 2.16-fold after oats supplementation. In addition, oats and tartary buckwheat significantly downregulated the expression of sterol regulatory element-binding protein 2 and peroxisome proliferator-activated receptors γ in liver tissue. In summary, our results suggested that oats and tartary buckwheat could modulate gut microbiota composition, improve lipid metabolism, and decrease oxidative stress and inflammatory responses in HFD fed rats. The present work could provide scientific evidence for developing coarse cereals-based functional food for preventing hyperlipidemia.

## 1. Introduction

Excessive intake of a high-fat diet (HFD) leads to abnormal lipid metabolism, which further gives rise to various chronic diseases, such as obesity, hyperlipidemia, and type 2 diabetes. By 2030, the number of people who are obese or overweight might reach 867.06 million in China [1], and obesity has become a major public health concern throughout the world. Meanwhile, gut microbiota and their metabolites such as short-chain fatty acids (SCFAs) impact the lipids and carbohydrate metabolism in the liver and adipose tissue, which is becoming a new target for anti-obesity treatment [2]. Thus, food intervention aiming at modulating the gut microbiota is available to improve metabolic health [3].

The gut microbiome has emerged as a key factor affecting human health and disease [4,5]. Elucidating the changes in gut microbiota composition has contributed to understanding the development and progress of obesity, metabolic syndrome, and type 2 diabetes mellitus [6]. The majority of gut microbiota serve as vital vehicles in the host metabolism by increasing energy harvest from the diet, which contributes to becoming overweight or obese [7]. In addition, microbiota-derived SCFA acetate can promote metabolic syndrome through regulating the gut-brain axis [8]. Numerous studies have reported that gut microbiome dysbiosis is directly or indirectly related to insulin resistance in type 2 diabetes mellitus [9]. Nevertheless, effective manipulation of the gut microbiota through diet has the potential to reduce metabolic diseases such as obesity and diabetes [10].

Epidemiological studies suggest that higher whole-grain cereals consumption reduces the risk of chronic diseases, including cardiovascular disease, type 2 diabetes, and certain types of cancer [11,12]. For instance, a barley kernel-based bread improved glucose metabolism that was associated with increased *Prevotella* abundance [13]. Previous studies reported that different varieties of coarse cereals reduced the blood glucose levels in rats with streptozotocin-induced hyperglycemia [14,15]. Buckwheat and foxtail millet could also improve lipid metabolism and blood glucose tolerance in vivo [16,17]. Moreover, our previous study suggested that the consumption of whole grain rice and wheat could regulate gut microbiota and improve SCFAs composition in rats [18].

Coarse cereals exert health-promoting benefits, which are largely due to their functional components such as dietary fiber, polyphenols, polysaccharides, and peptides [15,19,20]. Dietary fiber and phytochemicals in coarse cereals have the potential to regulate the gut microbiota, the balance of which is crucial to preserve gut homeostasis [21,22]. Furthermore, dietary fibers promote SCFAs production through intestinal bacterial fermentation [23]. Specifically, our recent research revealed that administration of oats increased gut microbiota diversity and the concentrations of SCFAs in normal rats [24]. In addition, tartary buckwheat protein markedly increased the SCFAs and the abundances of *Lactobacillus* and *Bifidobacterium* in HFD fed mice [25].

Oats, tartary buckwheat, and foxtail millet are common cereal grains in China. The impact of these three kinds of coarse cereal on gut health has gained great attention, but the comparative effects of them on gut microbiota and lipid metabolism remain unclear. Thus, the aim of this study was to determine the effects of these whole-grain cereals with matrix on the gut microflora and lipid metabolism in HFD fed rats.

## 2. Materials and Methods

### 2.1. Materials and Reagents

Oats (Variety name: Bayou No.1), tartary buckwheat (Variety name: Chuanqiao No.1), and foxtail millet (Variety name: Jingu No.21) were purchased from Hebei, Sichuan, and Shanxi Province, respectively. Main nutritional contents of cooked oats, tartary buckwheat and foxtail millet were shown in Table S1. The level of β-glucan was 4.19 ± 0.42 g/100 g in oats. The contents of total flavonoids and resistant starch were 1.76 ± 0.06 and 0.39 ± 0.01 g/100 g in tartary buckwheat, respectively. The levels of vitamin B1 was 0.27 ± 0.04 mg/100 g, and total dietary fiber content was 2.16 ± 0.02 g/100 g in foxtail millet. The diets containing oats, tartary buckwheat, and foxtail millet were ordered from Trophic Animal Feed High-tech Co. Ltd. (Jiangsu, China). All of the coarse cereals were cooked using a commercially available cooker as previously described [26]. All the cooked coarse cereals were ground prior to use in the experimental diets. Composition and energy density of the diets are presented in Table 1. Acetic, propionic, butyric, isobutyric, isovaleric, and valeric acid standards were purchased from Sigma-Aldrich Chemical Co. (Purity ≥ 99.5%, St. Louis, MO, USA). The primary antibody, anti-SREBP-2, was purchased from Santa Cruz Biotechnology (Dallas, TX, USA). All other antibodies were procured from Abcam (Cambridge, MA, USA).

### 2.2. Animals, Diets and Experimental Design

Sixty male Sprague Dawley rats (5 weeks old) were purchased from Vital River Laboratory Animal Technology Co., Ltd. (Beijing, China). After acclimating for 1 week, the rats were randomly divided into five groups (*n* = 12 per group, three per cage): a basal diet group (Con group), a high-fat diet group (HFD group), HFD containing 22% oat group (Oat group), HFD containing 22% tartary buckwheat group (Buc group), and HFD containing 22% foxtail millet group (Mil group). The initial body weight of all groups was almost the same, and the rats were treated with the aforementioned diets for 12 weeks. Food intake was monitored daily and body weight was measured every week throughout the experiment. The blood samples (5 mL) were collected by cardiac puncture, centrifuged at 4000 rpm for 10 min, and stored at −80 °C. Liver and epididymal fat were carefully collected, weighed, quickly frozen in liquid nitrogen, and stored at −80 °C for further study. Pancreas and colon were also collected and frozen in liquid nitrogen.

### 2.3. Biochemical Analysis in Serum

The levels of total triglyceride (TG), total cholesterol (TC), high-density lipoprotein cholesterol (HDL-C), low-density lipoprotein cholesterol (LDL-C), aspartate transaminase (AST), alanine aminotransferase (ALT), and free fatty acid (FFA) in serum were analyzed by the corresponding reagent kits (Zhongsheng Beikong Bioengineering Institute, Beijing, China). The levels of fasting glucose were determined using a glucometer (Johnson & Johnson, Milpitas, CA, USA).

### 2.4. Oral Glucose Tolerance Test (OGTT)

Rats were fasted 12-h overnight. Following that, OGTT was implemented 1 day prior to sacrifice. Then, gavage with glucose (2 g/kg body weight) was performed on rats. Tail blood was collected at 0, 30, 60, and 120 min using a glucometer. OGTT were calculated by the trapezoidal method and expressed as area under the curve (AUC) [27].

### 2.5. Histological Analysis

Liver, pancreas, epididymal adipose, and colon tissues were fixed in 10% formalin, embedded in paraffin, sliced into 4 μm thick sections, and then stained with hematoxylin-eosin (H&E). For the oil red O staining, frozen liver tissues were sliced and stained with oil red O. The stained sections were observed by using an optical microscope (Olympus, Tokyo, Japan). Pathology scoring was performed in a blinded manner by a pathologist in Peking University.

### 2.6. Western Blot Analysis

Protein samples of liver tissues were extracted, separated by sodium dodecyl sulfate-polyacrylamide gel electrophoresis, and loaded onto a nitrocellulose membrane according to our previous procedures [28]. The membranes were blocked with 5% defatted milk and then incubated with primary and secondary antibodies. Protein bands were visualized by fluorescence using an ODYSSEY FC Imaging System (LI-COR Biosciences, Lincoln, NE, USA).

### 2.7. Analysis of Cytokines and Antioxidant Status in Serum

The levels of tumor necrosis factor-alpha (TNF-α), interleukine-6 (IL-6), interleukin-1β (IL-1β), adiponectin, and insulin in serum and the concentration of zonula occludens-1 (ZO-1) in the colon were measured using ELISA kits (R&D Systems, Minneapolis, MN, USA). The levels of malondialdehyde (MDA) and total antioxidant capacity (T-AOC) and the activities of superoxide dismutase (SOD) in serum were determined with commercial kits (Nanjing Jiancheng Biological Technology Institute, Nanjing, China) following the manufacturer’s instructions.

### 2.8. Determination of Colonic SCFAs

Colonic content samples (80 mg, *n* = 6 per group) were mixed with 500 µL of methanol/water (*v/v* = 1:1) mixture and shaken by vortex for 30 min. Then, 50 µL supernatant that was collected after centrifugation (14,000 rpm, 10 min) was mixed with 50 µL of internal standard (5 µg/mL propionic acid-d2). Samples were derivatized with 50 µL 3-nitrophenylhydrazine (200 μM) for 30 min, and the supernatant was collected after centrifugation (14,000 rpm, 10 min). The resulting supernatant (5 µL) was analyzed on an Acquity UPLC I-Class system equipped with a BEH C18 chromatographic column (100 mm × 2.1 mm, 1.7 µm; Waters, Milford, MA, USA) coupled to a Xevo TQ-S mass spectrometer [29].

### 2.9. Gut Microbiota Analysis

Microbial DNA in colonic content samples was extracted according to the protocol described previously [18]. The V3-V4 hypervariable regions of 16S rRNA genes were amplified with primers 515F and 806R by a T100™ thermal cycler (Bio-Rad, Hercules, CA, USA). TruSeq DNA PCR-Free Library Preparation Kit (Illumina, San Diego, CA, USA) was used to construct the sequencing library. The sequencing was obtained from the Illumina NovaSeq 6000 platform at Novogene Bioinformatics Technology Co., Ltd. (Beijing, China). The Quantitative Insight into Microbial Ecology (QIIME) was used for further analysis. The sequences were classified into operational taxonomic units (OTUs) with 97% similarity. Then, the OTUs were subjected to taxonomy-based analysis by RDP Classifier (v2.13). Alpha diversity metrics and beta diversity were calculated using QIIME and displayed with R software. The principal coordinates analysis (PCoA), Venn diagram, and heatmap analysis of the major genus were performed by R software.

### 2.10. Statistical Analysis

All statistical analyses were performed using R 4.0.2. Continuous variables with normal distribution were presented as mean ± standard deviation (SD) and were performed by one-way ANOVA followed by Tukey’s post hoc multiple comparison test. Skewed distribution variables were indicated with P_50_ (P_25_, P_75_) and were examined by non-parametric statistical hypothesis test. Spearman’s correlation analysis between gut microbiota and biochemical parameters was performed and a heat map was generated. *p* < 0.05 was considered as statistically significant.

## 3. Results

### 3.1. Food Intake, Energy Intake, Body Weight, and Organ Weights

**Figure 1:**
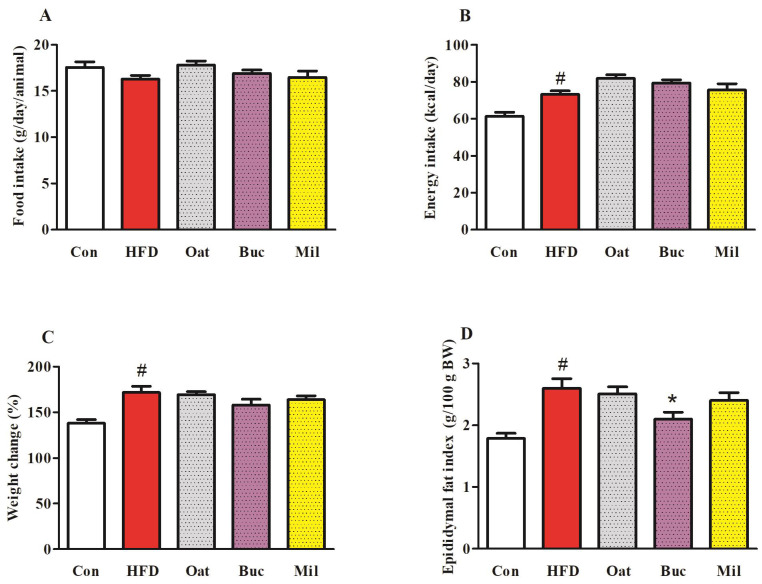
Effect of oats, tartary buckwheat, and foxtail millet supplementation on food intake (**A**), energy intake (**B**), percent change in body weight (**C**), and epididymal fat index (**D**) in high-fat diet fed rats. Con group, a basal diet group; HFD group, a high-fat diet group; Oat group, HFD containing 22% oat group; Buc group, HFD containing 22% tartary buckwheat group; Mil group, HFD containing 22% foxtail millet group. Data are presented as the mean ± SD (*n* = 12). # *p* < 0.05, compared to Con group, * *p* < 0.05, compared to HFD group.

There were no significant differences in food intake among the five groups of rats during 12 weeks (Figure 1A). Energy intake in HFD group was significantly increased compared to Con group (Figure 1B), while Oat, Buc, and Mil groups were not significantly different from HFD group. At the end of study, weight gain in the HFD group was significantly higher than in the Con group (Figure 1C), but weight gain in the Oat, Buc, and Mil groups was not significantly different from in the HFD group. The rats in the HFD group showed a 45.57% increase in epididymal fat index compared to Con group (Figure 1D). However, epididymal fat index was significantly lower in Buc group compared with the HFD group. The liver indexes among the five groups of rats were not significantly different (data not shown).

### 3.2. Lipid Metabolism-Related Parameters in Serum, Fasting Blood Glucose, and OGTT

**Figure 2:**
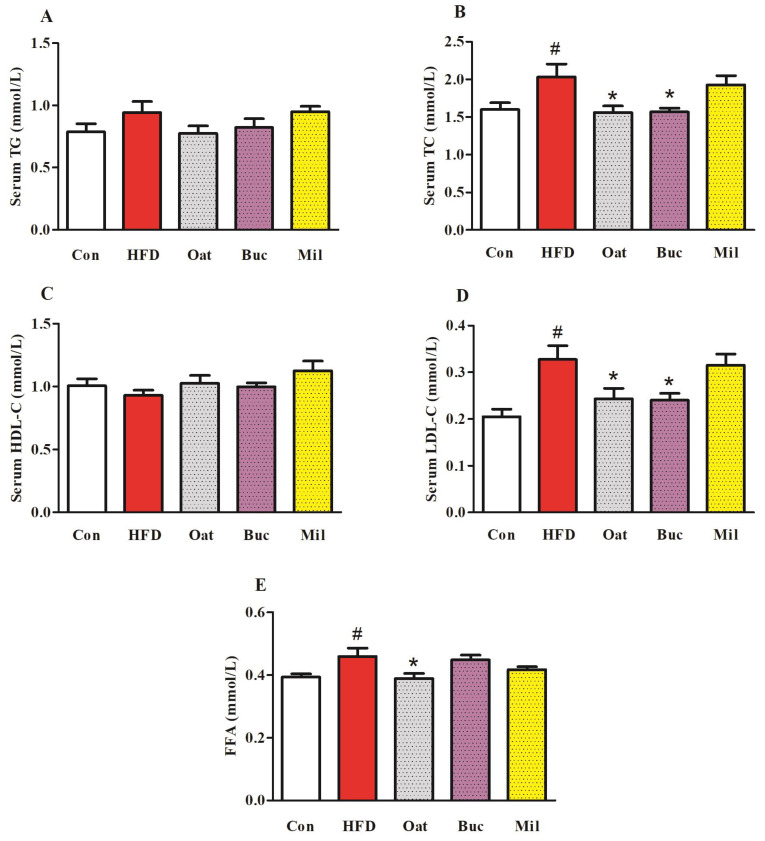
Effect of oats, tartary buckwheat, and foxtail millet supplementation on serum TG (**A**), TC (**B**), HDL-C (**C**), LDL-C (**D**), and FFA (**E**) in high-fat diet fed rats. Con group, a basal diet group; HFD group, a high-fat diet group; Oat group, HFD containing 22% oat group; Buc group, HFD containing 22% tartary buckwheat group; Mil group, HFD containing 22% foxtail millet group; TG, total triglyceride; TC, total cholesterol; HDL-C, high-density lipoprotein cholesterol; LDL-C, low-density lipoprotein cholesterol; FFA, free fatty acid. Data are presented as the mean ± SD (*n* = 12). # *p* < 0.05, compared to Con group, * *p* < 0.05, compared to HFD group.

**Figure 3:**
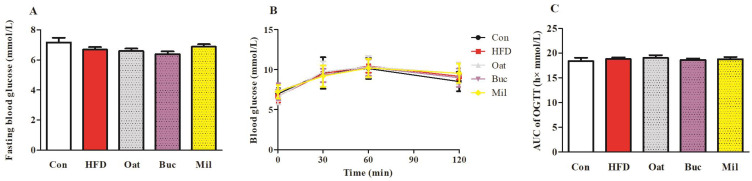
Effect of oats, tartary buckwheat, and foxtail millet supplementation on fasting blood glucose (**A**), OTGG (**B**), and AUC of OGTT (**C**) in high-fat diet fed rats. Con group, a basal diet group; HFD group, a high-fat diet group; Oat group, HFD containing 22% oat group; Buc group, HFD containing 22% tartary buckwheat group; Mil group, HFD containing 22% foxtail millet group; AUC, area under the curve; OGTT, oral glucose tolerance test. Data are presented as the mean ± SD (*n* = 12).

HFD feeding resulted in a significant increase in TC and LDL-C levels in serum (Figure 2B,D), but there were no significant differences in the TG and HDL-C levels in serum (Figure 2A,C) compared with the Con group. Serum TC and LDL-C in the Oat and Buc groups were remarkably decreased compared with that in the HFD group. There were no significant differences in the serum AST and ALT levels among the five groups (Figure S1). The HFD diet induced a higher level of FFA in the serum compared with the Con diet (Figure 2E), but the level of FFA concentration was significantly reduced in the Oat group compared with the HFD group. However, there were no significant differences in fasting blood glucose and AUC of OGTT among the five groups (Figure 3). 

### 3.3. Pro-Inflammatory Cytokine Levels, Antioxidant Capability, Adiponectin, and Insulin in Serum

**Figure 4:**
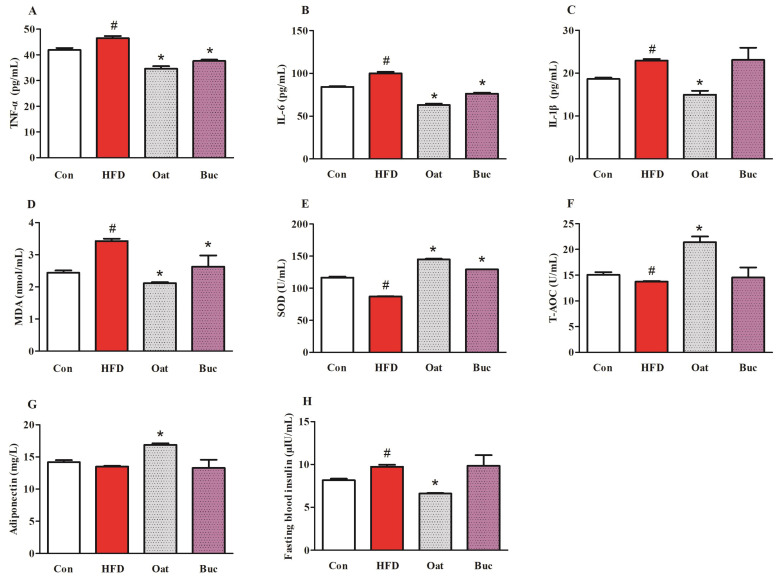
Effect of oats and tartary buckwheat supplementation on serum TNF-α (**A**), IL-6 (**B**), IL-1β (**C**), MDA (**D**), SOD (**E**), T-AOC (**F**), adiponectin (**G**), and fasting blood insulin (**H**) in high-fat diet fed rats. Con group, a basal diet group; HFD group, a high-fat diet group; Oat group, HFD containing 22% oat group; Buc group, HFD containing 22% tartary buckwheat group; TNF-α, tumor necrosis factor-alpha; IL-6, interleukine-6; IL-1β, interleukin-1β; MDA, malondialdehyde; SOD, superoxide dismutase; T-AOC, total antioxidant capacity. Data are presented as the mean ± SD (*n* = 10). # *p* < 0.05, compared to Con group, * *p* < 0.05, compared to HFD group.

Oat and Buc groups exerted beneficial effects on lipid metabolism-related parameters in serum (TC, LDL-C, and FFA), so serum pro-inflammatory cytokine levels, antioxidant capability, adiponectin and insulin, and colonic ZO-1 protein were measured for these two kinds of coarse cereal. In HFD group, the levels of TNF-α, IL-6, and IL-1β were significantly increased in serum, while oats supplementation significantly decreased their levels (Figure 4A–C). Moreover, TNF-α and IL-6 were significantly down-regulated in Buc group. HFD significantly reduced the activities of SOD and the concentrations of T-AOC, and increased the level of MDA in serum (Figure 4D–F), indicating that HFD diet decreased antioxidant capacity of the serum in rats. However, oat and tartary buckwheat supplementation significantly lowered MDA levels and increased SOD activities, and oats supplementation also significantly increased T-AOC when compared with the HFD treatment. Although HFD did not result in a significant decrease in adiponectin level, oats administration markedly increased adiponectin level (Figure 4G). In addition, fasting blood insulin was significantly higher in the HFD group compared with the Con group, but oats supplementation significantly reduced its level relative to the HFD group (Figure 4H).

### 3.4. Histological Analysis of the Liver, Pancreas, Epididymal Adipose, and Colon Tissues

**Figure 5:**
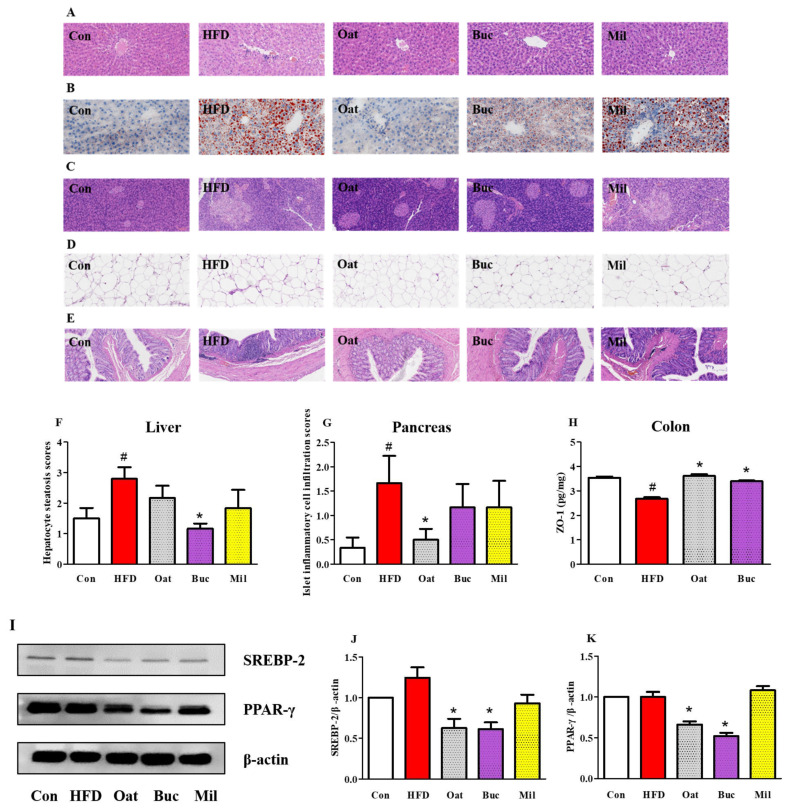
Effect of oats, tartary buckwheat, and foxtail millet supplementation on liver (**A**,**B**), pancreas (**C**), epididymal adipose (**D**), and colon (**E**) tissues in high-fat diet fed rats. Representative samples of liver tissue were stained with H&E (**A**) and oil red O (**B**). (**F**) Scores based on hepatocyte steatosis in the liver. (**G**) Scores based on inflammatory cell infiltration in the pancreas. (**H**) The protein expression levels of ZO-1 in the colon. (**I**) Western blot bands of SREBP-2 and PPAR-γ in the liver. (**J**,**K**) Densitometric analysis of SREBP-2 (**J**) and PPAR-γ (**K**) expressions relative to the loading control. Con group, a basal diet group; HFD group, a high-fat diet group; Oat group, HFD containing 22% oat group; Buc group, HFD containing 22% tartary buckwheat group; Mil group, HFD containing 22% foxtail millet group; ZO-1, zonula occludens-1; SREBP-2, sterol regulatory element-binding protein 2; PPAR-γ, peroxisome proliferator-activated receptors γ. Data are presented as the mean ± SD (*n* = 6). # *p* < 0.05, compared to Con group, * *p* < 0.05, compared to HFD group.

The histological analysis of the liver tissue showed that the liver steatosis induced by HFD was greatly attenuated following Buc supplementation (Figure 5A,B,F). An HFD diet induced inflammatory cell infiltration in the pancreas of rats, while oats supplementation significantly restored pancreatic islet morphology (Figure 5C,G). In line with the epididymal fat index shown in Figure 1D, the H&E staining of epididymal fat tissue revealed a drastically larger adipocyte size in HFD rats, while the oats and tartary buckwheat supplementation suppressed the increase of the adipocyte size (Figure 5D). Inflammatory cell infiltration in the colon can be observed in the HFD group, while oats supplementation lowered inflammatory cell infiltration (Figure 5E). Colonic ZO-1 protein expression in the HFD group was significantly lower than that in the Con group (Figure 5H), while ZO-1 protein was significantly higher in the Oat and Buc groups compared with the HFD group.

### 3.5. Expression of Proteins Involved in Lipid Metabolism

The expression levels of sterol regulatory element-binding protein 2 (SREBP-2) and peroxisome proliferator-activated receptors γ (PPAR-γ) in liver, which were involved in lipid metabolism, were not significantly changed in HFD group compared to Con group (Figure 5I–K). However, oats and tartary buckwheat supplementation significantly decreased the expression of these proteins. 

### 3.6. Coarse Cereals Supplementation Modulated the Gut Microbiota

**Figure 6:**
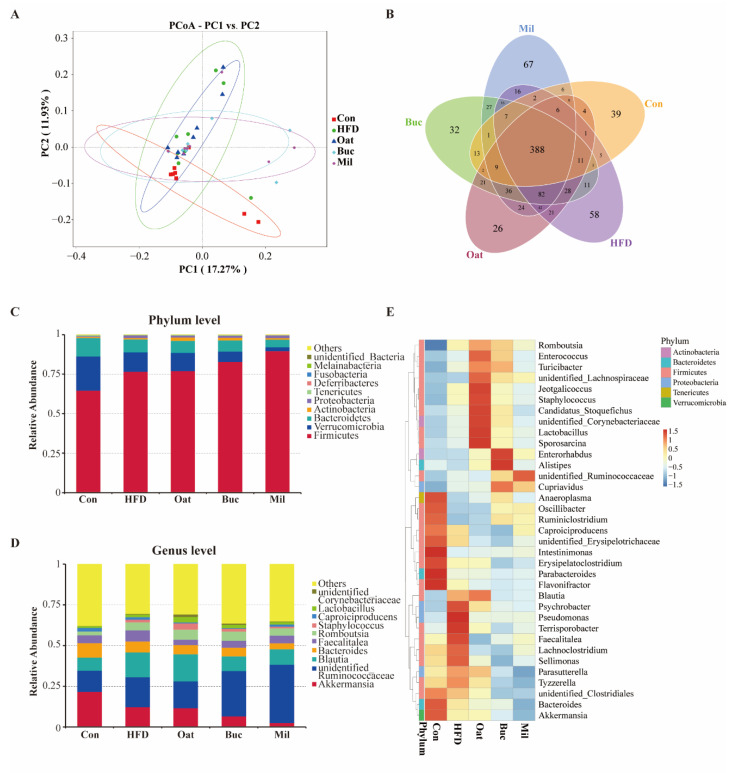
Effect of oats, tartary buckwheat, and foxtail millet supplementation on gut microbiota composition in high-fat diet fed rats. (**A**) Principal coordinate analysis (PCoA) of colonic microbiota (*n* = 8). (**B**) Venn diagram showing the shared and unique OTUs among the five groups. (**C**) Microbial community composition at the phylum level. (**D**) Microbial community composition at the genus level. (**E**) Heatmap of clustering analysis at the genus level. Con group, a basal diet group; HFD group, a high-fat diet group; Oat group, HFD containing 22% oat group; Buc group, HFD containing 22% tartary buckwheat group; Mil group, HFD containing 22% foxtail millet group.

HFD and coarse cereals supplementation has no significant effect on alpha diversity of colonic microbiota (data not shown). PCoA analysis demonstrated that it generated a distinct difference of microbial communities between HFD and Con groups (Figure 6A). A total of 388 OTUs (38.3%) were common among different groups (Figure 6B). The Mil group had more unique OTUs (67) than the Con (39), HFD (58), Oat (26), and Buc (32) groups. The influences of coarse cereals on the composition of gut microbiota at the phylum and genus levels was shown in Figure 6C,D. Furthermore, heatmap was used to show the abundance of 35 key genera of different groups in order to evaluate the impact of coarse cereals supplementation on the bacterial community of HFD fed rats (Figure 6E). At the phylum level, Firmicutes, Verrucomicrobia, and Bacteroidetes were the absolute dominated taxa in all groups, and the relative abundance of Firmicutes was much higher in the HFD group compared with the Con group. Tartary buckwheat and foxtail millet supplementation increased the Firmicutes (8.21% and 16.94%, respectively) and decreased Verrucomicrobia (46.64% and 79.54%, respectively) compared with the HFD group. At the genus level, HFD increased the relative abundance of unidentified *Ruminococcaceae*, *Blautia*, *Faecalitalea*, *Romboutsia*, and *Staphylococcus*, and decreased the relative abundance of *Akkermansia* and *Bacteroides* compared with the Con group. Oats and tartary buckwheat significantly increased the abundance of *Lactobacillus* and *Romboutsia*, and tartary buckwheat and foxtail millet significantly decreased the abundance of *Akkermansia* and *Blautia*.

### 3.7. Coarse Cereals Supplementation Regulated the Specific Bacteria

**Figure 7:**
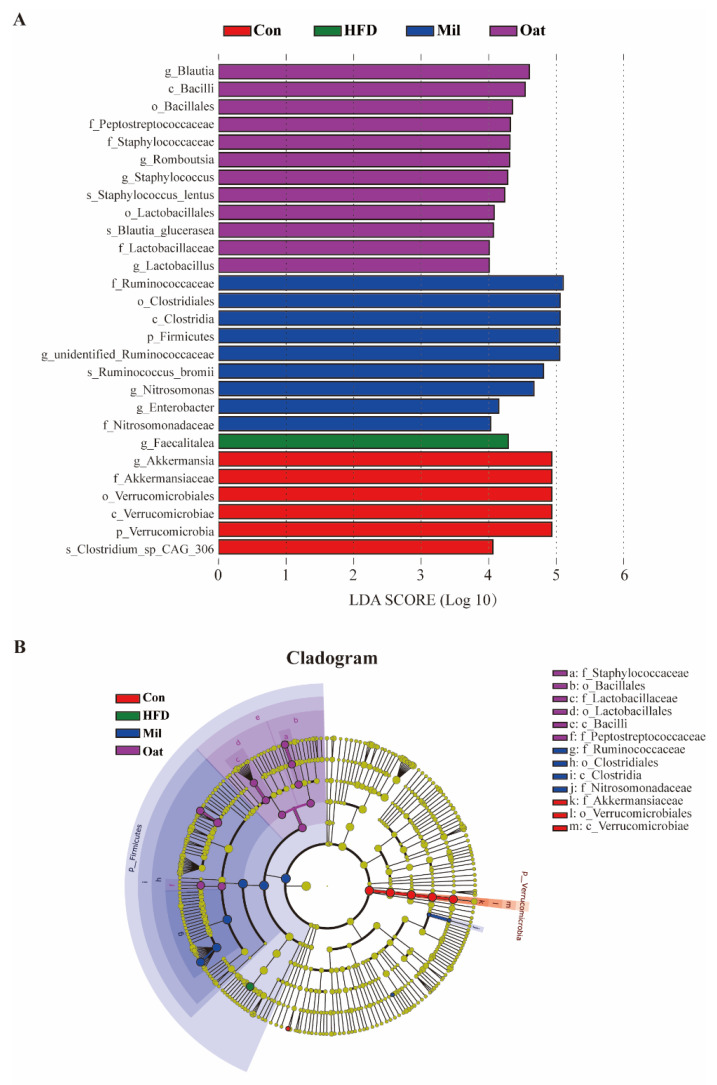
Linear discriminant analysis (LDA) effect size (LEfSe) analysis of the key genera of gut microbiota in high-fat diet fed rats. (**A**) Histogram of the LDA scores for differentially abundant bacterial taxa. (**B**) Cladogram visualizing the phylogenetic distribution of the gut microbiota community. Con group, a basal diet group; HFD group, a high-fat diet group; Oat group, HFD containing 22% oat group; Mil group, HFD containing 22% foxtail millet group; p, phylum; c, class; o, order; f, family; and g, genus.

Figure 7 showed the significant differences of gut microbiota in the taxa among five groups, including 9 genera. The Con group was dominated by *Akkermansia*, while the *Faecalitalea* was the most prevalent bacteria in the HFD group. The Oat group exhibited a predominance of *Blautia*, *Romboutsia*, *Staphylococcus*, and *Lactobacillus* at the genus level. The Mil group featured the genera unidentified *Ruminococcaceae*, *Nitrosomonas*, and *Enterobacter* at the genus level.

### 3.8. Correlation between Gut Microbiota and Pro-Inflammatory Cytokine, Antioxidant Capability and Lipid Metabolism-Related Indices

**Figure 8:**
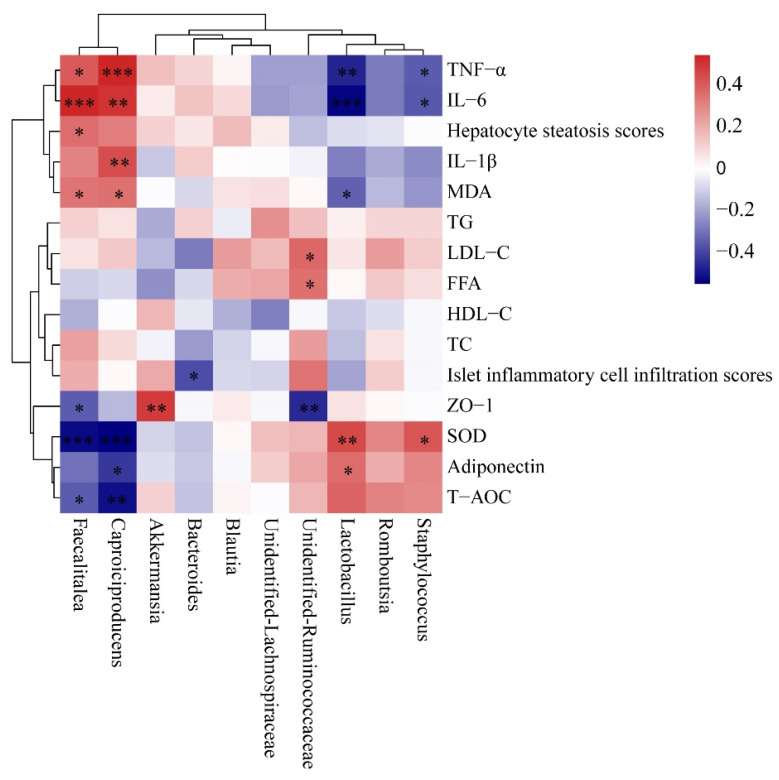
Heatmap of Spearman’s correlation between gut microbiota (top 10 at genus level) and pro-inflammatory cytokine, antioxidant capability, and lipid metabolism-related indices. * *p* < 0.05, ** *p* < 0.01, *** *p* < 0.001. TG, total triglyceride; TC, total cholesterol; HDL-C, high-density lipoprotein cholesterol; LDL-C, low-density lipoprotein cholesterol; FFA, free fatty acid; TNF-α, tumor necrosis factor-alpha; IL-6, interleukine-6; IL-1β, interleukin-1β; MDA, malondialdehyde; SOD, superoxide dismutase; T-AOC, total antioxidant capacity; ZO-1, zonula occludens-1.

Spearman’s correlation analysis showed that *Lactobacillus* abundance was positively associated with SOD and adiponectin, and negatively associated with TNF-α, IL-6 and MDA (Figure 8). *Akkermansia* abundance was positively associated with ZO-1, but *Faecalitalea* and unidentified *Ruminococcaceae* was negatively associated with ZO-1. *Staphylococcus* was negatively associated with TNF-α and IL-6 and positively associated with SOD. Unidentified *Ruminococcaceae* abundance was positively associated with LDL-C and FFA. *Faecalitalea* and *Caproiciproducens* abundance was positively correlated with TNF-α, IL-6, and MDA, but negatively correlated with SOD and T-AOC. 

### 3.9. Coarse Cereals Supplementation Regulated the SCFAs Production

There were no significant differences in SCFAs production between Con and HFD groups (Table 2). However, butyrate concentration was significantly increased by oat supplementation when compared with the HFD treatment. In Oat and Buc groups, the butyrate level was elevated by 2.16- and 1.77-fold when compared with the HFD group, respectively.

## 4. Discussion

In this study, intake of oat and tartary buckwheat attenuated oxidative stress, inflammatory responses, and hyperlipidemia in serum, downregulated the expression of lipid metabolism-related protein in liver, and increased the relative abundance of *Lactobacillus* and *Romboutsia* in colonic digesta of HFD-induced rats. Spearman’s correlation analysis showed that the changed bacteria were strongly correlated with oxidative stress and inflammation-related parameters. Histological analysis also demonstrated that intake of tartary buckwheat reduced the excessive lipid droplet accumulation in the liver and inhibited lipid accumulation in epididymal adipose tissue. In previous studies, oat and tartary buckwheat consumption was associated with the cholesterol-lowering effect [30,31]. The increased concentrations of SCFAs and alterations in the gut microbiota probably have an important role in ameliorating dyslipidemia [18].

Coarse cereals have potential health benefits through modulating intestinal flora. *Lactobacillus*, *Bifidobacterium*, and *Akkermansia* have beneficial effects on metabolism, stimulating fatty acid oxidation and inhibiting lipoprotein lipase activity in HFD fed mice [32,33]. Intake of embryo-remaining oat rice enhanced the production of SCFAs and increased the abundance of *Bifidobacterium* and *Akkermansia* in HFD fed rats [34]. Tartary buckwheat consumption alleviated hyperlipidemia and gastritis by maintaining intestinal homeostasis [35,36] and markedly increased the abundances of *Lactobacillus*, *Blautia*, and *Akkermansia* in HFD fed mice [16]. Foxtail millet supplementation ameliorated colorectal cancer by the microbial metabolites (tryptophan metabolites and SCFAs) activating related gut receptors [37]. Whole barley decreased cholesterol accumulation and counteracted gut dysbiosis in obese mice [38]. In addition, dietary intake of mixture coarse cereals reduced fat accumulation, decreased serum lipids levels, and increased the abundances of *Lactobacillus* and *Bifidobacterium* in HFD fed mice [39]. To the best of our knowledge, few comparative studies on the effects of these three kinds of coarse cereal on gut microbiota and lipid metabolism in HFD fed rats.

In this study, *Lactobacillus* and *Staphylococcus* were thought to contribute to inhibiting inflammatory response, while the abundances of *Faecalitalea* and *Caproiciproducens* were positively correlated with levels of inflammatory factors. Furthermore, our work suggested that oat supplementation increased the production of butyrate, which was an important metabolite of gut microbiota because it promoted host intestinal barrier function and alleviated inflammation [40]. Gao et al., (2020) also showed that dietary oat fiber was conducive to produce SCFAs and increased the abundance of the gut bacteria that generated anti-inflammatory metabolites and improved gut barrier function in LDLR^−/−^ mice [41]. Moreover, quinoa and buckwheat protein-rich flours decreased the plasma TC and LDL-C and elevated the production of SCFAs in rats [42]. 

Coarse cereals are rich in functional active substances such as dietary fiber and phytochemicals, which are considered to be natural prebiotics. Epidemiological study demonstrated that cardiometabolic risk was inversely associated with dietary fiber intake [43]. In a randomized controlled trial, dietary fiber (oat bran, 30 g/d) supplement decreased blood pressure and modulated the gut microbiota in patients with essential hypertension [44]. In another clinical trial, supplementation with 3 g/day of oat β-glucan reduced LDL-C, TC, and non-HDL-C in mildly hypercholesterolemic subjects [45]. In addition, increased consumption of coarse cereals containing dietary fiber contributes to healthy functioning of the intestine. Oat β-glucan mitigated the inflammatory status in colon, enhanced colonic barrier function, and increased gut microbiota-derived SCFAs (butyrate) in vivo [46,47]. Tartary buckwheat-resistant starch improved intestinal health by regulating the gut microbiota composition and increasing the yield of SCFAs in HFD fed mice [48]. In this study, oat and tartary buckwheat supplementation were significantly increased the ZO-1 protein expression, indicating they improved colonic barrier function in HFD-induced rats. 

It is worth noting that purified fiber supplementation has no effect on gut microbiota diversity in previous study [49]. The health benefits of whole grains were probably because of the synergistic action of multiple components in grains [50]. For example, oat bran as a complex food matrix increased *Bifidobacteria* and SCFA production, rather than its main functional compounds such as β-glucan and polyphenols in an in vitro fermentation model [49]. Tartary buckwheat is rich in resistant starch and flavonoids, which may have synergistic effects on lipid metabolism and gut microbiota [19]. Sorghum polyphenols and fructooligosaccharides worked synergistically to increase the abundances of *Bifidobacterium* and *Lactobacillus* during in vitro fermentation [51]. 

In addition to dietary fiber, phytochemicals (phenolic acids, flavonoids, etc.) in coarse cereals also had the potential to combat common nutrition-related diseases including cardiovascular disease, diabetes, and obesity. Flavonoids from oat exhibited an anti-hyperlipidemic effect via regulating gut microbiota that increasing *Akkermansia* and decreasing *Blautia* in mice [52]. Polyphenols from foxtail millet bran and shell could remodel the gut microbiota to prevent tumor and atherosclerosis in mice, respectively [53,54]. Furthermore, vitexin as a millet-derived flavonoid suppressed HFD-induced brain oxidative stress and inflammation by regulating gut microbiota in HFD fed mice [32]. 

There are some limitations of our study. First, the rodent gut microbiota differs from the human gut microbiota [55]. Second, the analysis of lipid metabolism-related protein expression is not enough, and relevant analysis is conducive to further elucidate the mechanism of anti-hyperlipidemic effect of coarse cereals. Third, the synergistic health benefits of functional components in coarse cereals such as dietary fiber and polyphenols is unclear. Further studies are needed to investigate cereal dietary fiber and polyphenols synergistic alleviating obesity via regulating gut microbiota.

## 5. Conclusions

Consumption of coarse cereals such as oats and tartary buckwheat was able to improve lipid metabolism and modulate gut microbiota composition. Oats and tartary buckwheat increased the abundance of *Lactobacillus* and *Romboutsia*, which was strongly correlated with anti-oxidant and anti-inflammatory effects. The colonic level of SCFAs such as butyrate was significantly increased after oats supplementation. This study can provide scientific evidence for the development of cereal-based functional foods.

## Figures and Tables

**Table 1:** Composition and energy density of the diets.

	Con	HFD	HFD + Oat	HFD + Buc	HFD + Mil
Oats	0	0	220	0	0
Tartary buckwheat	0	0	0	220	0
Foxtail millet	0	0	0	0	220
Casein	190	242	206	202	213
Corn starch	504	193	25	38	11
Dextrin	81	103	103	103	103
Sucrose	95	121	121	121	121
Soybean oil	24	30	30	30	30
Lard	16	195	183	187	186
Cellulose	48	61	51	41	56
Mineral mixture	27	36	35	34	35
Vitamin mixture	10	12	12	12	12
L-Cystine	3	4	4	4	4
Choline chloride	2	3	3	3	3
TBHQ	0.008	0.045	0.045	0.045	0.045
Total	1000	1000	993	995	994
Content of coarse cereals/%	0	0	22.2	22.1	22.1
Energy density (Kcal/g)	3.5	4.5	4.6	4.7	4.6
Protein (% energy)	19	19	19	19	19
Carbohydrate (% energy)	71	36	36	36	36
Fat (% energy)	10	45	45	45	45

**Table 2:** Effects of oats, tartary buckwheat, and foxtail millet supplementation on SCFAs in colonic contents of rats at the end of 12 weeks.

	Con	HFD	Oat	Buc	Mil
Acetic acid (μg/g)	44.22 ± 7.11	69.71 ± 18.29	76.17 ± 7.685	74.89 ± 13.54	66.82 ± 10.55
Propionic acid (μg/g)	22.41 ± 4.69	34.56± 8.99	41.28 ± 4.72	34.50 ± 6.02	33.42 ± 4.90
Butyric acid (μg/g)	16.77 ± 3.01 ^a^	15.69 ± 1.98 ^a^	33.92 ± 6.97 ^b^	27.71 ± 3.49 ^a,b^	15.96 ± 1.75 ^a^
Isobutyric acid (μg/g)	5.20 ± 0.77	7.30 ± 1.25	8.56 ± 0.76	7.31 ± 0.84	6.90 ± 0.89
Isovaleric acid (μg/g)	4.19± 0.59	4.97 ± 0.60	5.85 ± 1.05	4.60 ± 0.63	4.43 ± 0.37
Valeric acid (μg/g)	5.08 ± 0.86	7.52 ± 1.43	9.31 ± 1.37	7.12 ± 1.03	6.74 ± 0.66
Total SCFAs (μg/g)	104.25 (71.95, 120.45)	126.82 (72.34, 223.53)	163.67 (141.09, 206.58)	159.76 (95.48, 212.05)	127.03 (99.15, 163.81)

The values are the means ± SD or P_50_ (P_25_, P_75_). Con group, a basal diet group; HFD group, a high-fat diet group; Oat group, HFD containing 22% oat group; Buc group, HFD containing 22% tartary buckwheat group; Mil group, HFD containing 22% foxtail millet group. Mean values not sharing the same superscript letter (a and b) within a row are significantly different at *p* < 0.05 in a post hoc test.

## Data Availability

Data presented in this study are available on request from the corresponding author.

## Supplementary Materials

The following supporting information can be downloaded at: https://www.mdpi.com/article/10.3390/nu14132760/s1. Figure S1: Effect of oats, tartary buckwheat and foxtail millet supplementation on serum AST (A) and ALT (B) in high-fat diet fed rats. Con group, a basal diet group; HFD group, a high-fat diet group; Oat group, HFD containing 22% oat group; Buc group, HFD containing 22% tartary buckwheat group; Mil group, HFD containing 22% foxtail millet group; AST, aspartate aminotransferase; ALT, alanine aminotransferase. Data are presented as the mean ± SD (*n* = 12); Table S1: Main nutritional contents of cooked oats, tartary buckwheat and foxtail millet.
