# Supplementary material for: Effects of Oats, Tartary Buckwheat, and Foxtail Millet Supplementation on Lipid Metabolism, Oxido-Inflammatory Responses, Gut Microbiota, and Colonic SCFA Composition in High-Fat Diet Fed Rats
Source: Nutrients. 2022 Jul 4;14(13):2760. doi: 10.3390/nu14132760 (PMC9268892; doi:10.3390/nu14132760)
Supplement: Supplementary file 1 [file nutrients-14-02760-s001.zip › nutrients-1745395-supplementary.pdf]

Table S1. Main nutritional contents of cooked oats, tartary buckwheat and foxtail millet.

	Crude fat（g/100 g）	Crude protein（g/100 g）	Crude fiber（g/100 g）	Crude ash（g/100 g）	Moisture content（g/100 g）
Oats	5.51±0.05	14.66±0.11	4.26±0.42	1.90±0.00	6.42±0.06
Tartary buckwheat	3.76±0.01	16.13±0.14	8.78±1.00	2.56±0.01	6.48±0.04
Foxtail millet	4.35±0.29	11.87±0.02	2.04±0.20	1.19±0.02	7.42±0.03

Figure S1

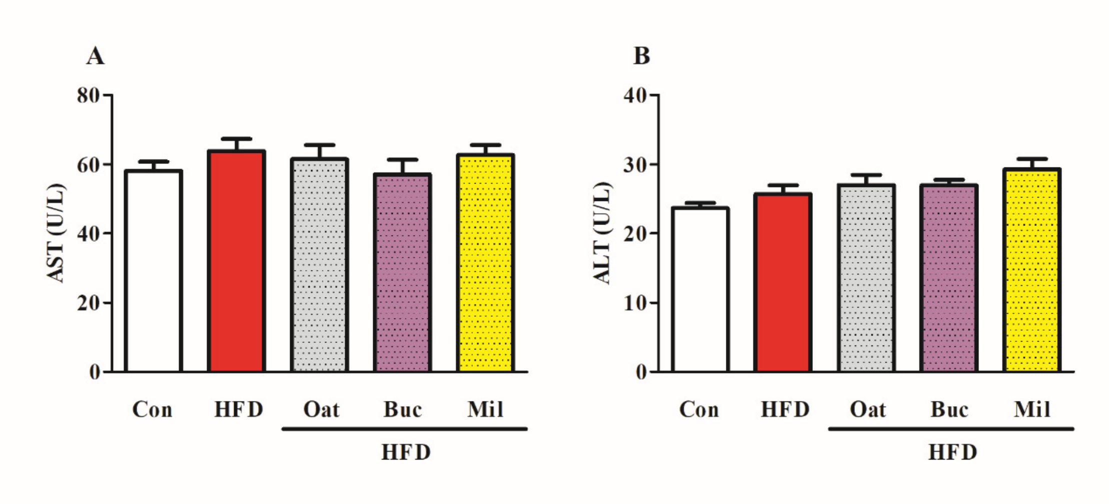

Figure S1. Effect of oats, tartary buckwheat and foxtail millet supplementation on serum AST (A) and ALT (B) in high-fat diet fed rats. Con group, a basal diet group; HFD group, a high-fat diet group; Oat group, HFD containing 22% oat group; Buc group, HFD containing 22% tartary buckwheat group; Mil group, HFD containing 22% foxtail millet group; AST, aspartate aminotransferase; ALT, alanine aminotransferase. Data are presented as the mean ± SD (n = 12).
